# Comparison of Gastric Insufflation Volume Between Ambu AuraGain and ProSeal Laryngeal Mask Airway Using Ultrasonography in Patients Undergoing General Anesthesia: A Randomized Controlled Trial

**DOI:** 10.7759/cureus.27888

**Published:** 2022-08-11

**Authors:** Adethen Gunasekaran, Kirthiha Govindaraj, Suman Lata Gupta, Stalin Vinayagam, Sandeep Kumar Mishra

**Affiliations:** 1 Anesthesiology and Critical Care, Jawaharlal Institute of Postgraduate Medical Education & Research, Puducherry, IND

**Keywords:** gastric antrum, intubation, ultrasonography, positive pressure ventilation, aspiration pneumonia, general anaesthesia

## Abstract

Introduction: Ambu AuraGain and ProSeal laryngeal mask airway are second-generation supraglottic airway devices (SADs) with added advantage of gastric drain and better oropharyngeal sealing pressure. The primary objective was to study the difference in the gastric insufflation volume between Ambu AuraGain and ProSeal LMA in patients undergoing general anesthesia.

Methods: This randomized controlled trial involving 120 adult patients scheduled under general anesthesia were randomized into either Ambu AuraGain or LMA ProSeal group. Gastric cross-sectional area was measured using ultrasonography at baseline, after mask ventilation, and at the end of surgery. Gastric volume was calculated from the measured cross-sectional area. Oropharyngeal sealing pressure, peak airway pressure, and postoperative complications were noted. Statistical analysis was done using SPSS version 22 (Armonk, NY: IBM Corp.) and p < 0.05 was considered statistically significant.

Results: Demographic profile of the study groups was comparable. There was a significant difference in gastric volume between the groups at the end of surgery with 5.91 ml (±9.68 ml) in Ambu AuraGain group and 12.28 ml (±13.05 ml) in the LMA ProSeal group (p = 0.001). Similarly, there was a difference in volume between baseline and at the end of the surgery within the groups also (Ambu AuraGain group, p=0.0012; LMA ProSeal group, p=0.0015, respectively). Though the oropharyngeal sealing pressure and peak airway pressures were comparable, increased incidence of postoperative complications was observed with LMA ProSeal.

Conclusion: Thus, Ambu AuraGain resulted in a lower gastric insufflation volume than LMA ProSeal with lesser incidence of postoperative complications.

## Introduction

Supraglottic airway devices (SADs) are commonly used in elective and emergency surgeries as they provide better airway protection with or without positive pressure ventilation [1]. These are less invasive than endotracheal intubation and are positioned outside the larynx [2]. Ambu AuraGain was launched as a new second-generation supraglottic airway device with characteristics like an inbuilt port for gastric drainage, preformed shape, and larger size of the cuff that forms the excellent seal around the pharynx [1,3]. Aspiration pneumonia remains a severe anesthetic complication and accounts for about 9% of all anesthesia-related deaths [4]. Second-generation SAD prevents gastric contents aspiration and thereby associated pulmonary complications [3]. Traditionally, oropharyngeal sealing pressure provides insight into the risk of gastric insufflation and aspiration risk [3,5-7]. Measuring the gastric cross-sectional area by ultrasonography to calculate the gastric volume can help predict the risk of aspiration in these patients [8-12]. Hence this study was planned to compare the gastric insufflation volume between Ambu AuraGain and ProSeal laryngeal mask airway (LMA) and its relationship with the oropharyngeal sealing pressure and the incidence of postoperative complications.

## Materials and methods

After obtaining approval from the institutional ethical committee (#JIP/IEC/2018/0167) and registering in the clinical trial registry, India (CTRI/2019/02/017753), this randomized control trial was conducted between March 2019 and August 2020 in a tertiary care center. All patients between the age group of 18 and 60 years, American Society of Anesthesiologists (ASA) physical status 1 and 2, with a surgical duration of approximately 2-4 h scheduled for elective surgery under general anesthesia, were included in this study. Patients with upper gastrointestinal problems, full stomach, known history of gastro-oesophageal reflux disorder, anticipated difficult mask ventilation or intubation, and pregnant patients were excluded from the study. About 126 patients satisfying inclusion criteria were enrolled in this study and randomized into either the Ambu AuraGain group (n=63) or the ProSeal LMA group (n=63) using computer-generated block randomization table using variable block sizes. Allocation concealment was done using the sequentially numbered opaque sealed envelope (SNOSE) technique. After obtaining informed consent from patients, fasting status and premedications were advised per institute protocol. On the day of surgery, standard anesthetic monitors were attached to the operation theatre. Baseline gastric antral cross-sectional area (CSA) was measured by an anaesthesiologist trained in point of care ultrasound, using 2-5MHz curvilinear ultrasound probeTM (SonoSite Ultrasound Machine; Bothell, WA: Sonosite S-ICUbered Opaque Inc.) placed over epigastrium in supine position with the left lobe of the liver, aorta, and inferior vena cava as a landmark. The probe's position was marked on the skin using the removable skin marker so that the same site could be used for measurement after the respective supraglottic airway device was placed.

After adequate preoxygenation, intravenous (IV) induction was initiated with fentanyl 2 μg/kg and propofol 2 mg/kg. Facemask ventilation (FMV) was initiated soon after the loss of verbal response and vecuronium 0.1 mg/kg was given and mask ventilation continued for 3 min. Depending upon the group allocation, an appropriate size SAD was inserted by an anaesthesiologist trained for at least one year and the cuff was inflated according to the size of the device. Intracuff pressure was measured with a calibrated aneroid manometer (Ambu cuff pressure gauge; Sula, Germany: VBM Medizintechnik GmbH) and was kept below 60 cm H_2_O. This was done by an anaesthesiologist who was unaware of the ultrasound findings. Insertion time was measured in seconds and counted from the time of opening the jaw to the appearance of the capnography waveform. Appropriate placement of SAD and ventilation was determined by an adequate chest rise, observing appropriate expired tidal volume and appearance of square waveform in capnography. Appropriate size Ryle's tube was inserted and the distal end was kept open. Ventilatory parameters were Vt 6-8 ml/kg ideal body weight, respiratory rate 12-14/min to maintain the EtCO_2_ of 35-40 mmHg, I:E ratio 1:2, pmax < 30 cm H_2_O. If the appropriate placement could not be achieved even after three attempts, then those patients were intubated and excluded from the study. Oropharyngeal sealing pressure (OSP) was measured after confirming the appropriate position of SAD and then switched over to bag and mask ventilation with the adjustable pressure limiting (APL) valve fully closed with fresh gas flow kept at 3 l/min. The pressure at which the palpable leak occurs was noted as oropharyngeal sealing pressure. Anesthesia was maintained with isoflurane, air, and oxygen mixture to the target minimum alveolar concentration (MAC 1-1.2) depending upon the vitals and the routine dose of muscle relaxants. Airway and ventilatory parameters such as oxygen saturation (SpO_2_), end-tidal carbon dioxide (EtCO_2_), peak airway pressure, and expired tidal volume were noted during FMV and positive pressure ventilation (PPV) with SAD. Measurement of gastric antral CSA in supine position was done after FMV before LMA insertion and at the end of surgery before LMA removal by using a 2-5 MHz curvilinear ultrasound probe (Bothell, WA: Sonosite S-ICUbered Opaque Inc.). Gastric cross-sectional area of the antrum in the supine position was determined based on formula using two perpendicular diameters - anteroposterior diameter (AP) and craniocaudal (CC) diameter by the following formula: CSA = (AP × CC × π)/4. Based on the antral CSA, total gastric volume (GV) was calculated using the formula: gastric volume (ml) = 27.0 + 14.6 × right - lateral CSA - 1.28 × age [12-14]. Postoperative complications such as nausea, vomiting, and sore throat were also noted.

Based on Park et al.'s study, with the estimated difference of about 25% in oropharyngeal leak pressure between two devices, sample size (n) was calculated as 63 patients in each group with a minimum expected clinical difference in Ambu AuraGain group with 95% confidence interval and 80% power using software Open Epi Version 3.01 [5]. The distribution of data on age, height, and weight were expressed as mean with standard deviation and were analyzed using an independent t-test. Oropharyngeal sealing pressures and the time taken to insert SAD were analyzed using an independent t-test. Gastric antral CSA and cuff pressure were analyzed using repeated measures ANOVA within the group and using Mann-Whitney ‘U’ test between the groups. All the statistical analysis was carried out at 5% level of significance and a p-value <0.05 was considered significant.

## Results

**Figure 1:**
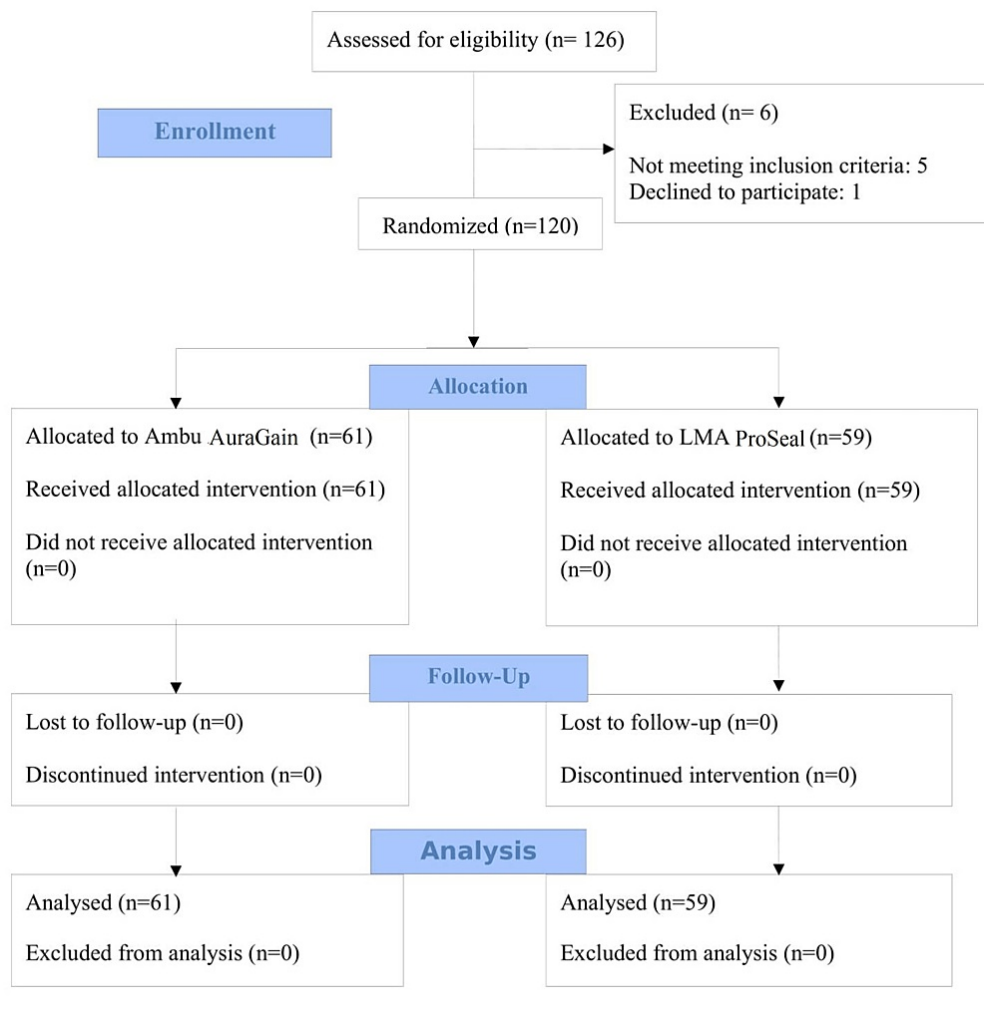
CONSORT diagram representing patient recruitment and allocation CONSORT: Consolidated Standards of Reporting Trials

The data from 120 patients were collected and statistical analysis was done (Figure 1). The mean of all demographic parameters like height, weight, body mass index, and sex and American Society of Anaesthesiologists (ASA) class was comparable between the groups (Table 1).

**Table 1:** Demographic parameters between Ambu AuraGain and LMA ProSeal group Age, height, weight, and BMI were represented in mean (SD-standard deviation) and sex; ASA classification was expressed in frequency and percentage (%). LMA: laryngeal mask airway; ASA: American Society of Anaesthesiologists

Parameters	Ambu AuraGain (n=61)	LMA ProSeal (n=59)
Age	48.2 (± 10.4)	46.7(± 12.2)
Height (cm)	162.8 (± 6.3)	161.6 (± 6.1)
Weight (kg)	60.6 (± 7.2)	60.4 (±7.6)
Body mass index (kg/m^2^)	22.9 (± 2.2)	23.1 (± 2.1)
Sex, male:female	30:31	30:29
ASA 1	22 (36.1%)	22 (37.3%)
ASA 2	39 (63.9%)	37 (62.7%)

There was no significant difference in gastric cross-sectional area among the groups at the baseline (p=0.875) and after mask ventilation (p=0.969), but a significant difference was noted at the end-operative period (p=0.026). A statistically significant difference was observed between baseline and end-operative values in both Ambu AuraGain group (p=0.013) and LMA ProSeal group (p=0.015) (Table 2).

**Table 2:** Comparison of gastric antral cross-sectional area before and after mask ventilation and at the end-op between Ambu AuraGain and LMA ProSeal group *P<0.05 is statistically significant. LMA: laryngeal mask airway; CSA: cross-sectional area

Groups	Antral CSA (cm)			p-Value
	Baseline	Post mask ventilation	End-operative period	
Ambu AuraGain (n=61)	1.67 (± 0.63)	1.68 (± 0.44)	2.46 (± 0.33)	0.013*
LMA ProSeal (n=59)	1.67 (± 0.62)	1.75(± 0.53)	3.83 (± 0.51)	0.015*
p-Value	0.875	0.969	0.026*	-

Significant difference was observed in gastric volume between the groups at the end of surgery, i.e., Ambu AuraGain group 5.91 ml (± 9.68) and LMA ProSeal group 12.28 ml (± 13.05) with the p=0.001. Similarly, gastric volume increased significantly at the end-op period as compared to baseline in both groups (Table 3). Oropharyngeal sealing pressure and peak airway pressure were comparable between both groups (Table 4).

**Table 3:** Comparison of gastric volume before and after mask ventilation and at the end-operative between Ambu AuraGain and LMA ProSeal group *P<0.05 is statistically significant. LMA: laryngeal mask airway

Groups	Mean gastric volume (ml)			p-Value
	Baseline	Post mask ventilation	End-operative period	
Ambu AuraGain (n=61)	1.68 (± 1.57)	2.43 (± 10.06)	5.91 (± 9.68)	0.0012*
LMA ProSeal (n=59)	1.75 (± 1.62)	5.45 (± 8.89)	12.28 (± 13.05)	0.0015*
p-Value	0.230	0.06	0.001*	-

**Table 4:** Comparison of oropharyngeal sealing pressure and peak airway pressure between Ambu AuraGain and LMA ProSeal groups LMA: laryngeal mask airway

Parameter	Ambu AuraGain (n=61)	LMA ProSeal (n=59)	p-Value
Oropharyngeal sealing pressure			
Baseline	28.39 (± 1.58)	28.49 (± 1.81)	0.753
End of surgery	28.23 (± 1.69)	28.32 (± 1.93)	0.781
Peak airway pressure			
Baseline	20.75 (± 1.58)	20.71 (± 1.58)	0.884
1st hour	20.70 (± 1.94)	20.67 (± 1.57)	0.916
2nd hour	20.60 (1.45)	21.04 (1.10)	0.097
End of surgery	21.0 (1.52)	21.63 (1.62)	0.421

In Ambu AuraGain group, 12 patients developed postoperative complications, of which, nine had a sore throat and three had nausea/vomiting. Whereas in LMA ProSeal group, 28 had postoperative complications, of which, 14 had a sore throat and 14 had nausea/vomiting. And there was a statistically significant difference with a p-value of 0.005 among the two groups (Table 5).

**Table 5:** Comparison of postoperative complications between Ambu AuraGain and LMA ProSeal groups *P<0.05 is statistically significant. LMA: laryngeal mask airway

Parameters	Ambu AuraGain (n=61)	LMA ProSeal (n=59)	p-Value
Nausea and vomiting	3 (4.9%)	14 (23.7%)	0.005*
Sore throat	9 (14.75%)	14 (23.7%)	0.005*
Total	12 (19.67%)	28 (47.4%)	-

## Discussion

In our study, the gastric insufflation volume was found to be lower in the Ambu AuraGain group compared to the LMA ProSeal group. Though both the LMA’s used in our study belong to the second-generation device with the provision for gastric drain, Ambu AuraGain provided better sealing with lower gastric volume following positive pressure ventilation compared to LMA ProSeal group at the end of surgery. Nonetheless, the oropharyngeal sealing pressure and peak airway pressure were comparable among both the groups with more incidence of postoperative complications of about 47.4% in the LMA ProSeal group and 19.6% in the Ambu AuraGain group.

SADs have replaced tracheal intubation and still, it is in evolving stage unless the risk associated with its usage is reduced. The risk of incomplete airway sealing, resulting in gastric insufflation; and also inflation of airways at pressures more than 20 cm H_2_O can result in the opening of esophageal sphincter [6]. However, these adverse outcomes were overcome by the invention of drainage tube for gastric decompression, thus reducing the risk of pulmonary aspiration. Following its development from the prototypical classic LMA, its added advantages made it possible to use even as an airway adjunct in cardiac arrest and during difficult airway scenarios [2]. The method to prevent aspiration while using SADs is by measuring the antral cross-sectional area by ultrasonography and calculating the gastric volume from it, which also plays a significant role in detecting the risk. Thus it is a basic bedside procedure that every anesthetist should be aware of. As described in a study conducted by Cieslak et al., though patients were fasted as per the ASA fasting guidelines, USG measurement done at baseline before induction revealed that the gastric volume was more in 21% of the patients, of which, 9% of the patient had change in the airway management plan [15]. Thus point-of-care measurement of gastric volume as a baseline parameter for all patients makes it unremarkable to save the patient from aspiration and further pulmonary complications. And also, its role in positive pressure ventilation is still questionable in certain circumstances as it was not a definitive airway. But a study by Ye et al. clearly defined that there was no difference in the antral cross-sectional area with second-generation SADs when compared with tracheal intubation during laparoscopic surgeries even in the Trendelenburg position [7]. This makes second-generation SADs equally placed on par with tracheal intubation. Thus, it can be used for both spontaneous and controlled ventilation. And the point-of-care ultrasound can be used to calculate the gastric volume as described in the methodology and aspiration of gastric contents can be anticipated earlier as done in our study. In our study, none of the patients had change in the airway management plan, and also gastric antral cross-sectional area was comparable between both the groups; whereas the gastric volume was found to be significant between the groups at the end of surgery (LMA ProSeal>Ambu AuraGain) and also within each group at two different time points, i.e., after mask ventilation and at the end of surgery (p<0.001). But very few studies have been done so far to assess the gastric volume after LMA insertion with positive pressure ventilation. This makes our study more unique when compared to the other studies.

Antral measurement can be done in supine and right lateral decubitus position using point-of-care USG. As described by Perlas et al., right lateral decubitus position makes the antrum more dependent and prominent thus resulting in accurate measurement [13]. The other method described by Shariffuddin et al. was gastric aspirate rather than gastric volume measurement by USG and they found that the gastric volume aspirated with Ambu AuraGain was less and not statistically significant, which was not done in our study [16]. Though the main limitation of SAD is the presence of unprotected airway with the leading risk of aspiration which can be prevented by its correct placement, i.e., its acts as a barrier at the level of upper oesophageal sphincter, and the incidence of aspiration was similar to that of tracheal intubation approximately 0.02% [7,17,18]. However, the antral cross-sectional area was measured in all three LMA’s namely LMA supreme, I-gel, and tracheal intubation by Ye et al., and there was no difference, but they failed to find out the gastric volume which helps us to anticipate aspiration following its usage; this is well marked in our study. And in our study, measured gastric volume was lesser in Ambu AuraGain group than LMA ProSeal group [7].

The other important parameter was oropharyngeal leak pressure (OLP) which indicates airway protection, and the methods used to quantify were audible noise detection, oral capnography, stethoscopic noise, and manometric stability respectively. Though the safety profile of different LMAs was evaluated extensively, still studies were ongoing to identify which device offers superior OLP [6]. The OLP was comparable between the Ambu AuraGain group and LMA ProSeal group in our study indicating that both the LMA devices were equally protective in offering airway protection; no other studies were available. At least 15 cm H_2_O of peak airway pressure was required to maintain adequate ventilation during face mask ventilation and the incidence of gastric insufflation occurs with inspiratory pressure reaches the threshold of 20 cm H_2_O during unprotected airway ventilation [17]. A study by Bouvet et al. inferred that 15 cm H_2_O provided the best balance between the probability of sufficient pulmonary ventilation and also the absence of gastric insufflation. But this was done in non-obese and non-curarized patients with FMV pressure-controlled ventilation [19]. Whereas Qian et al. proved more than 12 cm H_2_O was required to cause gastric insufflation in children [20]. Our study was conducted in adult patients, which showed peak airway pressures within the range of 20.73-21.38 cm H_2_O from the baseline (pre-LMA insertion) till the end of surgery at four different time points in both the groups without any significant difference among them, and we found that there was less association between OLP and gastric insufflation in both the groups. Reduced incidence of postoperative complications in Ambu AuraGain group makes it more efficient enough to be used in patients with shorter duration surgical procedures and lesser side effect profile.

The limitations of our study include gastric volume measurement which was done only at the start and end of surgery can be improved by serial hourly measurement and also non-objective depth of anesthesia assessment. Further, oropharyngeal leak pressure with the changes in the head position and the fibreoptic visualization of the proper placement of LMA and its provision as a guide for intubation was not evaluated in our study, which can be done in future studies.

## Conclusions

Thus, point-of-care ultrasonography can be used as a bedside tool for the assessment of gastric volume and aspiration risk by measuring the gastric volume derived from antral cross-sectional area. Though the gastric volume was not significant enough to cause aspiration in both the groups, it was significantly lower in Ambu AuraGain group than LMA ProSeal group, making the Ambu AuraGain superior with the lesser side effect profile.
